# Biocatalysis in Water or in Non-Conventional Media? Adding the CO_2_ Production for the Debate

**DOI:** 10.3390/molecules28186452

**Published:** 2023-09-06

**Authors:** Pablo Domínguez de María, Selin Kara, Fabrice Gallou

**Affiliations:** 1 Sustainable Momentum SL, Av. Ansite 3, 4-6, 35011 Las Palmas de Gran Canaria, Spain; 2 Department of Biological and Chemical Engineering, Aarhus University, Gustav Wieds Vej 10, 8000 Aarhus, Denmark; 3 Institute of Technical Chemistry, Leibniz University Hannover, Callinstr. 5, 30167 Hannover, Germany; 4 Chemical and Analytical Development, Novartis Pharma AG, 4056 Basel, Switzerland

**Keywords:** green chemistry metrics, biocatalysis, wastewater, CO_2_ production

## Abstract

Biocatalysis can be applied in aqueous media and in different non-aqueous solutions (non-conventional media). Water is a safe solvent, yet many synthesis-wise interesting substrates cannot be dissolved in aqueous solutions, and thus low concentrations are often applied. Conversely, non-conventional media may enable higher substrate loadings but at the cost of using (fossil-based) organic solvents. This paper determines the CO_2_ production—expressed as kg CO_2_·kg product^−1^—of generic biotransformations in water and non-conventional media, assessing both the upstream and the downstream. The key to reaching a diminished environmental footprint is the type of wastewater treatment to be implemented. If the used chemicals enable a conventional (mild) wastewater treatment, the production of CO_2_ is limited. If other (pre)treatments for the wastewater are needed to eliminate hazardous chemicals and solvents, higher environmental impacts can be expected (based on CO_2_ production). Water media for biocatalysis are more sustainable during the upstream unit—the biocatalytic step—than non-conventional systems. However, processes with aqueous media often need to incorporate extractive solvents during the downstream processing. Both strategies result in comparable CO_2_ production if extractive solvents are recycled at least 1–2 times. Under these conditions, a generic industrial biotransformation at 100 g L^−1^ loading would produce 15–25 kg CO_2_·kg product^−1^ regardless of the applied media.

## 1. Introduction

Biocatalysis is considered one of the pillars on which the future of sustainable chemistry will be based, following the green chemistry principles [1,2]. The reasons for this are that enzymes are natural (biodegradable) catalysts and can be synthesized from biogenic resources. The role of enzyme catalysis that contributes to fulfilling a broad number of the United Nations’ Sustainable Development goals has been recently addressed, strengthening their potential for future sustainable chemical syntheses [3,4]. Traditional considerations of green technology for biocatalysis, however, have often been based on qualitative estimations—“biocatalysis is green per se”. In challenging these generic thoughts, the need for quantitative environmental metrics to sustain these statements has been recently emphasized [5,6,7,8,9]. To that end, different metrics (mass- and energy-based) to assess the actual sustainability of a chemical process have been proposed by several research groups and industries (e.g., process mass intensity, atom economy, carbon efficiency, etc.) [6,8,10,11,12,13,14]. Among them, in particular, the E-Factor, developed by Prof. Sheldon decades ago, represents a very intuitive and rapidly implemented metric to assess the waste formation in a reaction (or in a part of it). Thus, the E-Factor, namely, kilograms of waste produced by a kilogram of product, provides hints on the (un)sustainability of a given reaction and enables rapid incorporation of mitigation measurements to improve the environmental burden of a process [5,6]. However, further in-depth analysis of the sustainability, in particular, related to the “quality” of the waste, is commonly needed to assess not only the quantitative production of waste but also its ultimate potential impact on the environment. Very recently, the option of converting all waste streams into CO_2_ equivalent production has emerged, assuming that, sooner or later, wastes will be converted to CO_2_ and released into the media. Thus, the introduced “Total Carbon Dioxide Release” (TCR) concept addresses the kilograms of CO_2_ produced by a kilogram of product [15]. The methodology was introduced at Novartis about a decade ago to support the quest to assess sustainable alternatives to classical chemistry conducted in fossil-derived solvents. The aim was to take more interest in an informed decision when it came to assessing water-based alternatives. The methodology represents a very straightforward and meaningful tool to compare produced wastes and evaluate the real impact of a reaction by using *the same currency* for all produced wastes. Thus, rather than waste effluent being benchmarked regardless of its mass production, it is benchmarked based on its ultimate CO_2_ formation. TCR in biocatalysis has been recently used to assess gate-to-gate processes [16] or to provide data on the CO_2_ contribution generated using solvents, including the transportation burden [17].

Biocatalysis is a rather versatile field in which different reaction conditions can be set. Enzymes can be used as isolated catalysts (once purified) and can be immobilized to enable their reuse for several cycles, and also in a continuous fashion. Likewise, whole cells containing overexpressing enzymes can be used as biocatalysts, too. The choice of one or another option depends on many factors, as thoroughly discussed recently [18]. With respect to the reaction media, enzymatic processes can be conducted in aqueous solutions—the traditional media for biocatalysis, inspired by natural reactions—but also in non-aqueous systems, the so-called non-conventional media, where bulk water is absent. The non-conventional options may include a broad range of systems, comprising solvent-free processes where the media is the substrate, micro-aqueous solutions (containing non-bulk quantities of water) or the use of supercritical fluids or neoteric solvents like ionic liquids or deep eutectic solvents, to cite some relevant examples combining biocatalysis and non-aqueous systems [18]. As a matter of fact, both strategies for biocatalysis—aqueous and non-conventional—may present pros and cons for their implementation. On the one hand, water is broadly recognized as a non-hazardous solvent and, moreover, it is the natural environment for enzymes in life processes. On that basis, many researchers have proposed its use as the paradigm for a green solvent. Furthermore, recent combinations of enzymes with other chemo-catalysts in water have re-emphasized their use for industrial purposes [19,20,21,22,23]. The downsides of water, however, such as the low solubility of many relevant molecules—which force the use of organic cosolvents—and the generation of large volumes of wastewater, have been systematically pinpointed [8,24]. On the other hand, non-conventional media enable the dissolution of higher substrate loadings, leading to intensified processes that can be closer to industrial interests (e.g., high substrate loadings, ease of connecting reaction steps in an industrial environment by using organic solvents, etc.) [8,24,25,26,27]. However, many enzymes deactivate in non-aqueous media and, furthermore, introducing fossil resources (e.g., solvents) may compromise the ecological footprint of the biocatalytic reaction. Herein, the introduction of renewable-based solvents can ameliorate that impact partially (based on the CO_2_ neutrality of them), provided that the solvent synthesis is aligned with green chemistry principles as well [16]. Despite the broad use of both strategies in biocatalysis—with outstanding examples also at an industrial scale—studies providing quantitative metrics that may determine (and compare) the real environmental impact of enzymatic reactions in aqueous or in non-conventional media have not been reported hitherto. These assessments are needed to substantiate the green claims with which biocatalysis is traditionally associated [7,8]. Therefore, to assess all options in detail, herein a discussion based on different process scenarios for enzymatic reactions both in aqueous and non-conventional media will be provided. The analysis will cover both parts of the reaction, the upstream (enzymatic synthesis) and the downstream (product purification) and will focus on the ultimate CO_2_ production of each system as the parameter to evaluate the quantitative sustainability of the processes.

## 2. Results and Discussion

To study the CO_2_ production of different reaction media employed in biocatalysis, a gate-to-gate strategy will be followed. Thus, it is assumed that all reagents, solvents and catalysts are already present in the chemical plant and that the purified product (after downstream) ends up at the gate of the chemical plant for commercialization [16]. In that way, the assessment calculates the environmental impact of the reaction: what is the actual consideration for the media choice, aqueous or non-conventional? It must be noted, though, that the location of the chemical plant, the previous synthesis and transportation of solvents and reagents and other (broader) factors (e.g., land use) need to be considered in further analyses if a complete, holistic assessment is required [17].

### 2.1. Using the Same Currency: Converting Waste Effluents into Carbon Dioxide Production

**Figure 1:**
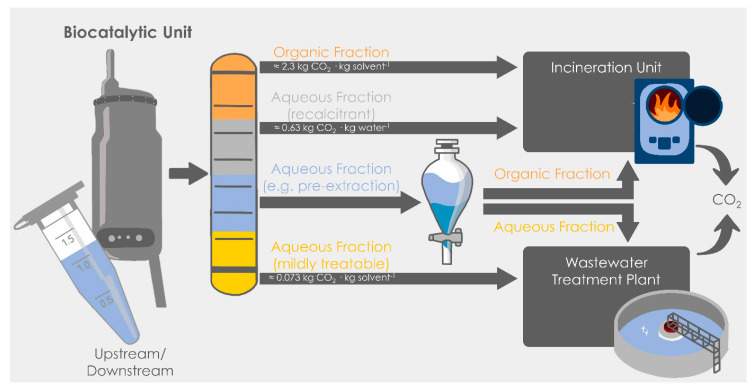
Overview of the waste effluents that a biocatalytic reaction may create (comprising both up and downstream) and ways of treatment depending on the waste quality. Data for CO_2_ production in the incineration and in the wastewater treatment units are taken from Krell et al. [28].

A given biocatalytic reaction involves generically two main steps, namely the upstream, where the actual enzymatic reaction takes place, and the downstream, where the formed product is purified into a marketable form [16]. The upstream is composed of a reaction media, either aqueous or organic, reagents and the (bio)catalysts, which can be in the form of whole-cells or isolated enzymes (both forms can be immobilized). The downstream part depends on the complexity of the product purification and the final market need. In some cases, products at technical grade are accepted at certain markets (e.g., bulk applications), which may simplify the downstream significantly. In some other cases, however, extremely high product purities are mandatory, as in the pharmaceutical industry. The downstream may then range from rather simple precipitation steps (with or without a final washing procedure to remove impurities) to extractive strategies, distillation, chromatography or crystallization, to cite some options. Overall, the biocatalytic process generates two primary waste effluents: an organic fraction (collecting all solvents) and a wastewater effluent (Figure 1).

The usual fate of the organic fraction—ideally after several reuses to improve sustainability and economics—is the incineration plant, where it will be converted into CO_2_ and released to the milieu. In the first-generation metrics developed at Novartis, based on a conservative and pragmatic scenario [11,28], a prototypical organic fraction would generate ~2.3 kg CO_2_·kg product^−1^ as the equivalent of incinerating a mixture of tetrahydrofuran–methanol–heptane (1:1:1 m/m/m). On the other hand, wastewater can be disposed in different processing plants, depending on the waste recalcitrance present in the water (Figure 1). The best scenario would be to submit the aqueous effluent to a traditional wastewater treatment plant, which after some conventional steps would be released into the environment as recycled water, with a minimal but still some CO_2_ associated production (nothing is for free). In this best-case scenario, the environmental impact would be in the range of ~0.073 kg CO_2_·kg product^−1^ (assuming effluents with a total organic carbon loading of 20 g kg^−1^ wastewater) [28]. In the worst-case option for the water, however, when recalcitrant wastewater is produced and mild treatments are not feasible, the incineration unit is the fate of that effluent, leading to a production of ~0.63 kg CO_2_·kg product^−1^. For a prototypical biocatalytic reaction, it may be expected that the wastewater treatment impact will possibly be in between the two cases. Thus, a pre-extraction step may be needed to remove cosolvents and chemicals below the required threshold. The produced organic fraction would be sent to the incineration unit, while the obtained wastewater would be then disposed in the mild treatment plant (Figure 1). In this case, the contribution of the pre-extraction step must be considered in the analysis of the environmental impact as well, incorporating two effluents, one organic for incineration and another aqueous for mild wastewater treatment (Figure 1). Overall, these quantitative estimations may enable practitioners and researchers to determine the actual CO_2_ production at an early stage and allow redirection of the research to options that may be less impactful for the milieu. This strategy to measure CO_2_ production can be further adapted to a case-by-case basis when real masses of chemicals and solvents (and their potential recalcitrance) are available for analysis.

### 2.2. The Water Hazard Class Concept (WGK)

**Figure 2:**
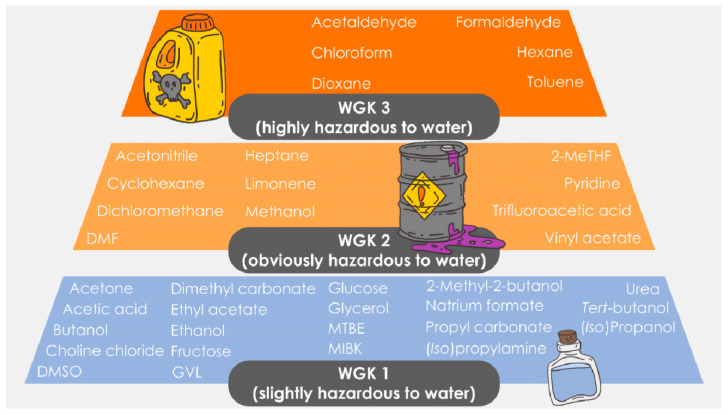
Water hazard classes (WGK) of different chemicals often used in biocatalysis as (co)solvents or reagents (data taken from Rigoletto database) [29].

As it can be observed, the key point to reaching a more or less sustainable option in biocatalysis is how wastewater can be treated, what ultimately depends on its composition and recalcitrance. Therefore, to have some preliminary assessments on how different chemicals may affect the wastewater treatment route (Figure 1), chemicals can be classified according to their *Wassergefährdungsklasse* (WGK or water hazard class), available at the Rigoletto database [29]. Thus, chemicals are divided into the groups “non-hazardous to water”, WGK 1 (“slightly hazardous to water”), WGK 2 (“obviously hazardous to water”) or WGK 3 (“highly hazardous to water”). In Figure 2, a selection of compounds commonly used in biocatalysis—as solvents, cosolvents or reagents—is presented within their respective WGK.

As observed, many (co)solvents and reagents used in biocatalysis fall within the WGK 1, enabling a certain use of chemical reactions in aqueous media. It must be noted, though, that chemicals must be kept below the threshold (to the bare minimum in fact, as a simple common-sense rule) if mild wastewater treatment procedures are to be established to reach lower CO_2_ production (Figure 1). Thus, it can be anticipated that possibly in most cases, a pre-extraction step to reduce concentrations below such thresholds will be needed [28], as discussed in the previous section (Figure 1), contributing to the final environmental impact. Likewise, the generation of by-products during the reaction—which can change its WGK level—cannot be underestimated and biodegradability assessments need to be performed in each individual case, considering by-product formation before deciding which wastewater treatment unit(s) are used [28].

### 2.3. Metrics Based on CO_2_ for Different Scenarios

Having the tools to calculate the CO_2_ production of the biocatalytic processes in hand, an in-depth discussion on the debate of aqueous vs. non-conventional media from an environmental perspective can be now established. An important parameter is the substrate solubility and its loading in the reactor, which depends on the media used. Since biocatalytic reactions are often performed in water, low substrate concentrations are commonly applied, because many organic molecules are sparingly soluble in aqueous media. The use of water-miscible cosolvents may assist in enabling better dissolutions of substrates (potentially at the cost of incorporating chemicals that will possibly need to be extracted before wastewater treatment, Figure 1). In non-conventional media, however, higher substrate loadings are shown from many examples reported in the literature. Therefore, the analysis for biocatalytic reactions must consider a broad range of substrate concentrations, starting from very diluted systems to more industrially sound concentrated reactors.

**Figure 3:**
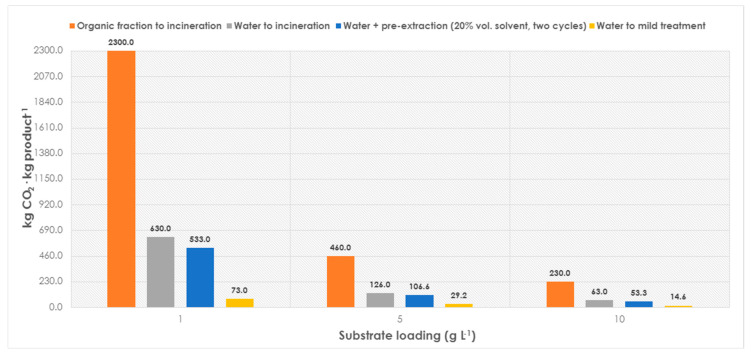
Production of CO_2_ of a biocatalytic reaction (upstream), with commonly used substrate loadings (1–10 g L^−1^), and different media: non-conventional (organic media), with recalcitrant water sent to incineration, with water with pre-extraction, or with water that can be mildly treated in conventional wastewater treatment plant.

Following those premises, in the first step of the assessment, the commonly reported biocatalytic conditions (low substrate loadings) were taken, i.e., 1–10 g substrate L^−1^ and four systems were considered: (i) organic media (which goes completely into incineration); (ii) recalcitrant water (which goes completely into incineration); (iii) effluent with a pre-extraction step using 2 × 10% (*v*/*v*) organic solvent (the organic part goes into incineration and the treated aqueous part into mild wastewater treatment); or (iv) a reaction with only wastewater to be mildly treated. The data on CO_2_ production associated with each case are depicted in Figure 3.

As it can be observed (Figure 3), the use of a non-conventional media—particularly at the low substrate loadings of 1 g L^−1^—penalizes dramatically the reaction, reaching up to more than 2 tons of CO_2_·kg product^−1^ if a single use of the solvent is considered. Even if an aqueous biotransformation associated with a mild wastewater treatment could be established, the CO_2_ production at such low substrate loadings (1 g L^−1^) would still result in an unacceptable outcome from a sustainability perspective (73 kg CO_2_·kg product^−1^). Assuming a more realistic scenario—with wastewater that would need some pre-extraction step(s)—and considering now higher substrate loadings, a standard biotransformation in water (10 g L^−1^) would generate 55–65 kg CO_2_·kg product^−1^ while the non-conventional media would render 230 kg CO_2_·kg product^−1^ (Figure 3). Overall, from the obtained data it appears that performing biocatalysis in aqueous media would result in a more environmentally friendly outcome than using non-conventional systems, where the use of an organic solvent is clearly an environmental burden. Reuse of the solvent would obviously improve this significantly.

**Figure 4:**
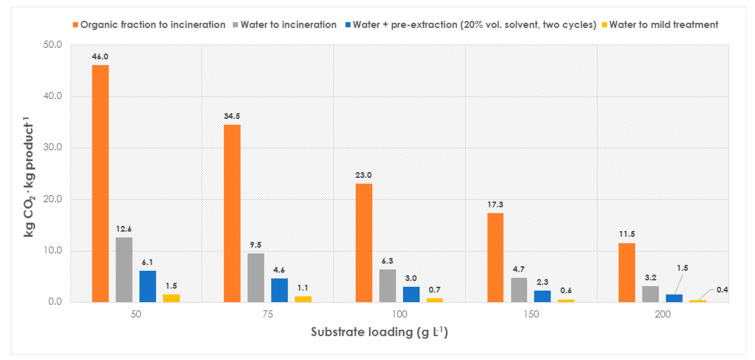
Production of CO_2_ of a biocatalytic reaction (upstream), with industrially sound substrate loadings (50–200 g L^−1^), and different media: non-conventional (organic media), with recalcitrant water disposed to incineration, with water with pre-extraction of chemicals, or with water that can be mildly treated in conventional wastewater treatment plants.

Subsequently, another range of substrate loadings (50–200 g L^−1^), closer to industrial interests, was assessed. Therein, the total CO_2_ production decreases considerably, since resources are more efficiently used (more substrate per solvent volume) [8,16]. As a clear conclusion, the need for process intensification appears mandatory to reach truly sustainable processes [25,30,31,32] (Figure 4).

As observed, an analogous trend is observed at higher substrate loadings (when compared to diluted systems, Figure 3), and biotransformations in water result in more environmentally friendly outcomes than processes in non-conventional media. Taking, for example, 100 g L^−1^ (the classic estimation as a rule-of-thumb to determine whether biotransformations can be potentially applied at an industrial scale), using an organic solvent as the reaction media would lead to a production of 23 kg CO_2_·kg product^−1^ (solvent recycling may obviously decrease the figures significantly). Notably, applying processes in aqueous media, with values in the range from 0.70 to 3 kg CO_2_·kg product^−1^, would be reached depending on the number of pre-extraction steps that the process would need (related to the use of hazardous chemicals based on the WGK, see Figure 1 and Figure 2). At higher substrate loadings (>100 g L^−1^), processes are clearly more sustainable, and less CO_2_ is produced because resources are more properly used.

**Figure 5:**
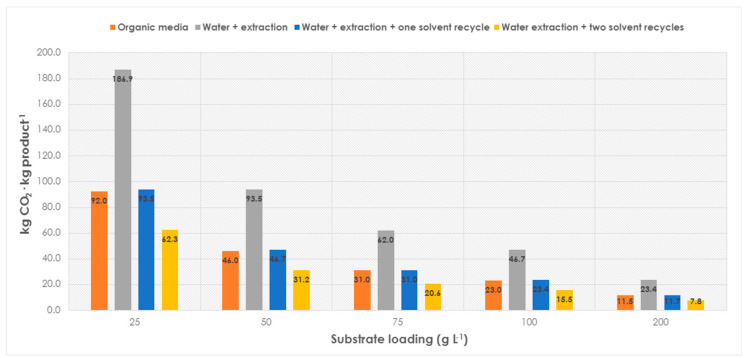
Production of CO_2_ in biotransformations at different substrate loadings (25–200 g L^−1^) and using either an organic media as solvent, or an aqueous solution to which an extraction (2X) with an organic solvent is applied, and subsequent mild wastewater treatment follows. The extractive solvent is used just once or recycled one or two times (assuming 10% loss per cycle). The environmental impact of distillation of the organic media (energy) for the downstream is not considered.

So far, the apparent conclusion would be that it is always better to conduct biotransformations in aqueous media, since non-conventional media options are more pollutant in terms of CO_2_ production, due to the burden associated with the ultimate solvent incineration (Figure 1). However, the presented data are related to the upstream part only, that is, the enzymatic synthesis of a certain molecule. To validate a complete biocatalytic reaction, the impact of the downstream unit must be considered as well [16]. While for biotransformations applied in organic media, the distillation of the solvent may work (with some energy used that would lead to more CO_2_ production, though), the downstream units applied to the biocatalytic aqueous reactions typically include an extractive step with organic solvents, normally twice the reactor volume (2X). Herein, recycling of the extractive solvents may be very relevant to reach improved values of CO_2_ production. The results are depicted in Figure 5.

When the downstream unit is incorporated into the analysis, the aqueous biotransformation shows a significantly higher environmental impact, due to the contribution of the organic solvent used for the extraction. Under those conditions, the use of a non-conventional media appears to be more sustainable than aqueous solutions (23 and 46.73 kg CO_2_·kg product^−1^, respectively, at 100 g L^−1^, Figure 5). Interestingly, the environmental impact of both approaches becomes comparable when the extractive solvent can be recycled at least one time (assuming a 10% solvent loss between each cycle). With one recycling step, the CO_2_ production results are analogous in water and non-aqueous media (23 vs. 23.46 kg CO_2_·kg product^−1^). If the extractive solvent can be recycled more than one time, performing biotransformations in water may become a more sustainable approach than processes conducted in non-conventional media (albeit solvent reuse, in this case, may also improve figures again). Overall, the results clearly emphasize the need to establish biotransformations with high substrate loadings and resource recycling to reach decent and low values of CO_2_ production while achieving industrial requirements simultaneously [8]. It must be noted that the time necessary to reach an optimized performance in a biocatalytic transformation is commonly a time-consuming challenge that makes such a scenario still relatively rare. Herein, performing biocatalysis in bulk water may usually become a straightforward option with reduced environmental impact. The use of non-edible, “dirty” fractions of water as reaction media may constitute an excellent option to set up more sustainable reactions without compromising the depletion of precious resources [21].

Finally, it must be noted that, in addition to what the environmental metrics dictate, when it comes to industrial applications, financial considerations are critical for the decision to go for one or other options, as sufficient margins are *in fine* necessary to ensure the sustainability of the business itself. Ultimate decisions are specific to the nature of the chemistry, the process and the actual composition of the waste streams. In general terms, organic waste can potentially be recycled, yet regulatory constraints have put the bar very high in terms of the required quality for the recycled solvents (on top of the investment required for the recovery process). Alternatively, incineration can bring some value back with energy recovered, but the inherent carbon release with the incineration process is costly and subject to taxes (that may likely increase in the future). The introduction of bio-based solvents may bring options in some cases. On the other hand, wastewater treatment costs may depend on the actual strategy followed—or needed, depending on the recalcitrance of the effluent—but in general terms, they may remain economically attractive and may be an alternative to fossil-derived solvents [28,33], provided that decent substrate loadings are set.

## 3. Materials and Methods

### Calculation of the TCR

Based on the recent literature [28], the total carbon dioxide release, expressed as kilograms of CO_2_ per kilogram of product, was calculated according to the following formulae:

TCR_org fraction_ = Kilograms of organic fraction × 2.3 (incineration unit).

TCR_aq. fraction_ = Kilograms of wastewater × 0.63 (incineration unit).

TCR_aq. fraction_ = Kilograms of wastewater × 0.073 (mild wastewater treatment).

Total TCR: TCR_1_ + TRC_2_ + … + TRC_n_ (several synthetic steps or units).

## 4. Concluding Remarks

Biocatalysis results in a broadly versatile tool and processes that can be implemented not only in aqueous solutions—the natural environment for enzymes—but also in the so-called non-conventional media such as the absence of bulk water. This paper has made use of the recently reported “Total Carbon Dioxide Release” (TCR) concept to quantitatively evaluate whether there are biocatalytic systems that may be more sustainable than others. When the upstream unit is evaluated separately, performing enzymatic reactions in aqueous media clearly lead to largely improved ecologic impacts when compared to reactions in non-aqueous media. The reason is the environmental burden that organic solvents may create while incinerated. However, when the downstream unit is incorporated into the assessment, the water media usually needs an extraction step (using an organic solvent) to purify the product. Under those conditions, the production of CO_2_ appears comparable if extractive solvents are recycled at least one time. In general and in estimative terms, industrial biotransformation at 100 g L^−1^ will produce 15–25 kg CO_2_·kg product^−1^ regardless of the applied media (aqueous or non-conventional). Herein, aspects related to the hazardousness of the used chemicals (e.g., WGK) and the need for pre-steps to purify the effluents are crucial because the incorporation of extra steps for wastewater treatment will enhance CO_2_ production as well. Overall, the final sustainability level will depend on the wastewater treatment to be applied. Thus, if mild treatment processes can be established, reactions in water will become a very attractive alternative for biocatalysis. Research in wastewater treatment strategies—also combined with biocatalytic steps that may purify effluents selectively—seems to be an important topic to be considered in future investigations.

## Data Availability

Not applicable.
